# Bounding pandemic spread by heat spread

**DOI:** 10.1007/s10665-022-10253-4

**Published:** 2023-01-06

**Authors:** Teddy Lazebnik, Uri Itai

**Affiliations:** 1 Department of Cancer Biology, Cancer Institute, University College London, London, UK; 2 TRST AI, Tel Aviv, Israel

**Keywords:** Diffusion rate boundary, Graph-based stochastic *SIR* model, Partial-knowledge pandemic management

## Abstract

The beginning of a pandemic is a crucial stage for policymakers. Proper management at this stage can reduce overall health and economical damage. However, knowledge about the pandemic is insufficient. Thus, the use of complex and sophisticated models is challenging. In this study, we propose analytical and stochastic heat spread-based boundaries for the pandemic spread as indicated by the Susceptible-Infected-Recovered (SIR) model. We study the spread of a pandemic on an interaction (social) graph as a diffusion and compared it with the stochastic SIR model. The proposed boundaries are not requiring accurate biological knowledge such as the SIR model does.

## Introduction

Over the history of mankind, pandemics cause repetitive catastrophic suffering [1]. It causes significant increase in the mortality rate [2], major economic losses [3], and substantial political instability [4]. However, proper management of the pandemic can significantly reduce all of this [5, 6]. Nonetheless, suitable governance during a pandemic time requires an understanding of the pandemic’s dynamics. Unfortunately, this task is very challenging. The main difficulty is the uncertainty in real time. To reduce this, one needs to consider all the relevant factors. Nevertheless, pointing out the suitable features that appear in real time is extremely hard [7]. The process of collecting epidemiological, clinical, and biological data is time-consuming, expensive, and complex at the operational level [8, 9]. In addition, policymakers need to act fast during the beginning of the pandemic to contain it at an early stage [10]. Inability to do so will result in greater disaster later in the pandemic [10].

Thus, providing policy-making with good analytic tools is essential. The fashion to obtain data-driven decisions is epidemiological-mathematical models [11]. These provide an analytical framework to obtain an analysis of the pandemic’s spread dynamics [12–14]. A large group of epidemiological models is based on the Susceptible-Infected-Recovered (SIR) model [7]. This model provides good baseline results [15]. The *SIR* model assumes that the course of an epidemic is short compared with the life of an individual. Therefore, the size of the population may be considered to be constant. This assumption is reasonable as far as it is not modified by deaths due to the epidemic disease itself. Furthermore, the *SIR* model assumes all individuals in the population are initially equally susceptible to the disease ($S$) and only one individual is infected ($I$) at the beginning of the pandemic. Moreover, it is further assumed that complete immunity is conferred by a single infection. In other words, it is possible to represent the *SIR* model using a system of non-linear ordinary differential equations where the average infected rate, $\beta$, and the average recovery rate, $\gamma$, are known:

1 $$\begin{aligned} \begin{array}{l} \frac{\mathrm{{d}}S(t)}{\mathrm{{d}}t} = - \beta S(t)I(t) \\ \\ \frac{\mathrm{{d}}I(t)}{\mathrm{{d}}t} = \beta S(t)I(t) - \gamma I(t) \\ \\ \frac{\mathrm{{d}}R(t)}{\mathrm{{d}}t} = \gamma I(t). \end{array} \end{aligned}$$

Naively, one would consider the average infected rate $\beta$ and the average recovery rate $\gamma$ to be deterministic quantities that might cause model artifacts. For example, a susceptible individual $(p \in S)$ can be infected and transformed into the infected sub-population $(I)$ in a given time $t$. Immediately afterward, in time $t+1$, there is a probability $\gamma$ that the same individual is recovered and transformed to the recovered sub-population ($R$) [16]. To overcome this, we considered these quantities to be stochastic. This is because the uncertain nature of multiple epidemiological, social, and economic processes produce these coefficients. Hence, it is possible to treat these coefficients as a transformation probability between the states [17].

To gain a more epidemiological detailed model, one can use an interaction graph to represent infection routes. From an epidemiological point of view, an interaction graph gives a more descriptive representation of infections between individuals [18]. Formally, an interaction graph is where individuals are the graph’s nodes and the graph’s edges are the possible infection routes. Indeed, Wang et al. [19] proposed a graph-based Susceptible-Infected-Susceptible (SIS) model. In their settings, each individual is represented as a node in a static, connected, and random graph. Similarly, Hau et al. [20] proposed an SEIR (E-exposed) model for sexually transmitted diseases. The authors defined the interactions between individuals using a bipartite static graph. These approaches are shown to well capture the pandemic spread dynamics. However, they still depend on a precise approximation of the infection and recovery rates [20]. This is due to the resilience problem in the ordinary differential equations [6]. Formally, we define an *infection graph* to be a graph $G := (V, E \subset V \times V)$ where $V$ are the nodes of the graph that represent individuals in a population with one of three epidemiological states (according to the SIR model’s definition) using a timed finite-state machine [21], and $E$ is the set of possible epidemiological interaction between individuals that can cause infection. For example, two individuals who work together in the same room have an edge between them as they can infect each other.

Another possible approach to tackle the pandemic spread prediction task is using heat spread. The transformation of heat on manifold plays an important role in many fields of science and engineering [22–24]. Heat spread shown to be promising in both theoretical [25, 26] and practical settings [27, 28]. The heat spread can be represented using the following partial differential equation:

2 $$\begin{aligned} \frac{ \partial u(t, \bar{x})}{ \partial t} = c \Delta u(t, \bar{x}), \end{aligned}$$

where $u: {\mathbb {R}}^{n+1} \rightarrow {\mathbb {R}}$ is a function, $t$ is the time, $\bar{x}$ is an $n$-dimensional space, and $c \in {\mathbb {R}}^+$ is the diffusion coefficient. The diffusion coefficient, $c$, can be treated as the average rate in which a physical area is heated. In our case, the average rate a pathogen is gathered inside an individual’s body. We note that the classical definition of the function $U$ is the temperature. However, additional interpolations can be applied. For instance, probability of the arrival of information. The second definition is spatially discrete compared to the proposed continuous definition proposed in Eq. (2). A discrete version of the heat spread equations takes the form:

3 $$\begin{aligned} \frac{ \partial u(t, \bar{x})}{ \partial t} = c \Sigma _{i=0}^{n} \frac{ \partial ^2 u(t, \bar{x})}{ \partial x_{i}^2} \end{aligned}$$

such that

$$\begin{aligned} \frac{ \partial u(t, \bar{x})}{ \partial t} := \frac{u(t+h, \bar{x}) - u(t, \bar{x})}{h} \quad \end{aligned}$$

and

$$\begin{aligned} \quad \frac{ \partial u(t, \bar{x})}{ \partial x_{i}} := \frac{u(t, [x_1, \dots , x_i + h, \dots x_n]) - u(t, [x_1, \dots , x_i, \dots x_n])}{h}, \end{aligned}$$

where $$ h \in {\mathbb {R}}^{+} \backslash \{0\}$$ [29].

Graphs are locally, on the node-level, isometric to manifold with a dimensional corresponding to the number of neighbors of the center node. Hence, assuming a graph $G := (V, E)$, the heat spread dynamics for each node $v \in V$ agrees with Eq. (3) such that $h = 1$ and $n = |\{v_i \in V \; | \; (v, v_i) \in E \}|$.

Following this, one can conclude that knowledge is required to obtain a fine approximation of the heat spread in an interaction graph. Specifically, only information on the interaction between individuals is needed. While the stochastic graph-based *SIR* model is based on more precise biological, social, and epidemiological knowledge, this information is not necessarily available during the beginning of a pandemic.

Thus, one can use the diffusion spread model, which requires less information and thus easier to approximate, to obtain an initial upper-bounded estimation to the pandemic spread compared to the SIR-based model. Nonetheless, as far as we aware of, no such comparison has been investigated so far. To fill this gap, we propose two upper boundaries for the pandemic spread in the population based on the heat spread coefficient. Our method is based on the heat spread on interaction graphs. This allows us to provide policymakers with a range of insights based on the connection between the two. This paper is organized as follows: in Sect. 2, we present two upper boundaries (*maximum* and *mean*) of a stochastic graph-based *SIR* model using the heat spread. In Sect. 3, we evaluate the usefulness of the proposed boundaries in a $k$-regular and random graphs. Following this, we evaluate the boundaries on social network data from Facebook to simulate realistic interaction graph settings. In Sect. 4, we discuss the possible epidemiological usage of these boundaries with their limitations and propose future work.

## Pandemic spread bounded by heat spread

To formalize the heat equation on a single node, one needs to calculate the probability of the node being *infected*. The probability a node $i$ with $|N_b(i)|$ adjacent nodes ($N_b(i)$ is the set of adjacent nodes to node $i$) would be infected is corresponding to the probability that each infected adjacent node ($v_j \in N_b(i)$) would infect node $i$.

4 $$\begin{aligned} p_i(\text {infected}) := 1 - \prod _{j \in N_b(i)} \big (1 - p_j(\text {infected}) \big ), \end{aligned}$$

such that $p_j(\text {infected}) = 0$ if node $j$ is not infected and some probability $p \in (0, 1]$ otherwise.

Based on these dynamics, we formally define the epidemiological interaction graph as follows. Let $G := (V, E)$ be a underacted, connected graph such that $E \subset V \times V$ and $|V| = N$. Each node $v \in V$ is representing an individual in the population. A node is defined by a finite-state machine with three states $\{S, I, R\}$—corresponding to the *SIR* model’s epidemiological states. In addition, the edge $e = (v_i, v_j) \in E$ is a possible interaction between two individual $v_i, v_j \in V$ such that $i \ne j$.

Following this, a stochastic *SIR* on an infection graph can be defined as follows. Given an infection graph $(G)$ and the parameters $\beta , \gamma \in (0, 1]$. At a given point in time, if $v_j \in N_b(v_i) \wedge v_j \in S \wedge v_i \in I$, than $v_j$ infected. Viz, $v_j$ transforms to state $I$ at a probability $\beta$. In addition, if $v_i \in I$ than $v_i$ recover. Namely, transforms to state $R$ at a probability $\gamma$. The process is terminated when $I$ reaches zero. Lazebnik et al. [16] had proved that the only recurrent state for the stochastic *SIR* model is $(S, I, R) = (N - d, 0, d)$ such that $1 \le d \le N$. Thus, the asymptotic state of the dynamics is achieved when $I = 0$. Therefore, the process halts.

Akin, one can define the heat spread on an infection graph as follows. Given an infection graph $(G)$ and the parameter $c \in {\mathbb {R}}^{+}$. At a given point in time, if $v_j \in N_b(v_i) \wedge v_j \in S \wedge v_i \in I$, then $v_j$ becomes infected. That is, $v_j$ transforms to state $I$ after $\lceil {\frac{1}{c}} \rceil$ time steps. Moreover, if $v_i \in I$ then $v_i$ recovered. Namely, transforms to state $R$ if $\forall v_j \in N_b(v_i)$ such that $v_j \in I$. The process is terminated when $I$ reaches zero. By treating the dynamics as a Markovian process [30], one can notice that the only recurrent state of the process takes the form $(S, I, R) = (0, 0, N)$. This happens because all individuals would eventually be infected and recover, assuming a connected graph. Hence, the asymptotic state of the dynamics is achieved when $I = 0$. Consequently, the process halts.

Based on these definitions, given an interaction graph that represents a population, one can bound the pandemic spread according to the stochastic *SIR* model using the heat spread model as shown in Theorem 1. In the following, we will show that the basic infection rate of the SIR model is dominated by the basic infection rate of the diffusion process.

### Theorem 1

Given an infection graph $(G)$ with infection rate $\beta \in (0, 1]$ and recovery rate $\gamma \in (0, 1]$. In addition, assuming the initial condition $(S, I, R) = (N - 1, 1, 0)$. Thus, exists a diffusion rate $c \in {\mathbb {R}}^{+}$ that agrees with:

5 $$\begin{aligned} \forall t \in {\mathbb {N}}: R_0^{\mathrm{{SIR}}(\beta , \gamma )}(t) \le R_0^{\mathrm{{Diffusion}}(c)}(t), \end{aligned}$$

where $R_0^{\mathrm{{SIR}}(\beta , \gamma )}(t)$ is the basic infection rate of the SIR model. Namely,

$$\begin{aligned}R_0^{\mathrm{{SIR}}(\beta , \gamma )}(t) := \max (0, \frac{R(t)-R(t-1)+I(t)-I(t-1)}{max(1, R(t)-R(t-1))}) \le \frac{\beta }{\gamma } I(t-1)\end{aligned}$$

for a graph-based SIR model with infection rate $\beta$ and recovery rate $\gamma$, and $R_0^{\mathrm{{Diffusion}}(c)}(t)$ is the basic infection rate of the diffusion model. I.e.,

$$\begin{aligned}R_0^{\mathrm{{Diffusion}}(c)}(t) := \max (0, \frac{R(t)-R(t-1)+I(t)-I(t-1)}{max(1, R(t)-R(t-1))}) \le c I(t-1)\end{aligned}$$

for a graph-based heat spread model with diffusion rate $c$. Of note, while the definitions of both $R_0$ metrics are identical when represented using the SIR’s model states (i.e., $S(t), I(t), R(t)$), they are not identical in practice due to the differences in the dynamics.

### Proof

Let $v_0$ be the node which satisfies $v \in I$ at $t = 0$. Node $v_0$ is a single node according to the assumptions. Performing a breadth-first search (BFS) [31] starting from $v_0$. During the BFS, each node $v \in G$ has been allocated with a distance $d$ from $v_0$. I.e., $d(v_0, v)$ is the length of the shortest path between $v_0$ and $v$ in the graph, $G$. On one hand, for the stochastic *SIR* process, the worst case scenario obtained where $\beta = 1$ and $\gamma = \epsilon > 0$. This happens as larger $\beta$ and smaller $\gamma$ increase the pandemic spread. In this case,

6 $$\begin{aligned} \begin{array}{l} R_0^{\mathrm{{SIR}}(\beta , \gamma )} \le R_0^{\mathrm{{SIR}}(1, \epsilon )} \le \max _{k \in [1, N-1]}(|\{v \in V \; | \; d(v_0, v_i) = k\}|). \end{array} \end{aligned}$$

Intuitively, $\max _{k \in [1, N-1]}(|\{v \in V \; | \; d(v_0, v) = k\}|)$ is the infection front of the graph as all nodes (individuals) in the graph that are neighboring an infected nodes are the largest set of individuals that can be infected in a single step in time. By setting the diffusion rate $c$ to be $\max _{k \in [1, N-1]}(|\{v \in V \; | \; d(v_0, v) = k\}|)$, for any infection rate $\beta \in (0, 1]$ and recovery rate $\gamma \in (0, 1]$, the condition

7 $$\begin{aligned} \forall t \in {\mathbb {N}}: R_0^{\mathrm{{SIR}}(\beta , \gamma )}(t) \le R_0^{\mathrm{{Diffusion}}(c)}(t), \end{aligned}$$

satisfied. $$\square $$

A corollary of Theorem 1 is that the pandemic spread and heat spread are isomorphic where $\beta = c = 1$ and $\gamma = 0$. This is true since, the processes are defined to be isomorphic if and only if $\forall t \in {\mathbb {N}}: |\{v \in V \; | \; v \in I\}|$ is identical for both processes. In addition, an isomorphism analysis between the two models is provided in the Appendix.

### Definition 2.1

The *event horizon* is the set of nodes $H$ which satisfies:

$$\begin{aligned} H := \{v_i \in V \; | \; i \ne j \wedge v_j \in N_b(v_i): v_j \in I \wedge v_i \in S\} \end{aligned}$$

Following this, one can point out that, at time $t=0$ in both processes the size of infected nodes depends on the interaction graph. For each step in time, the event horizon $H \subset V$ is infected, while the other nodes are not. This means both processes are deterministically identical for $\beta = c = 1$ and $\gamma = 0$.

While this boundary holds for any pandemic, we note that this boundary is not tied for the most realization of a pandemic. This is due to the high variance in the pandemic spread [11, 32, 33]. Therefore, one can bound the mean pandemic spread given the interaction graph, as shown in Theorem 2. The mean pandemic spread provides a more tied boundary of the pandemic spread given only the infection rate $\beta$.

### Theorem 2

Given an infection graph $(G)$ with infection rate $\beta \in (0, 1]$ and recovery rate $\gamma \in (0, 1]$. In addition, assuming the initial condition $(S, I, R) = (N-1, 1, 0)$. The vector of mean infection time ($V^i_j$) agrees with the minimal (e.g., if $x_j$ is another solution with $x_j \ge 0$ then $x_j \ge V^i_j$ ) non-negative solution of the following equation:

8 $$\begin{aligned} {\left\{ \begin{array}{ll} V^i_j = \frac{1}{\beta } + \Sigma _{k \ne j} \beta V^i_k, \; i \ne j \\ V^i_j = 0, \; \text {otherwise} \end{array}\right. }, \end{aligned}$$

where $V^i_j \in {\mathbb {N}} \cup \infty$ is a random variable that stands for the time pass that an infection that starts at individual $i$ will infect individual $j$. We define the *“hitting time”* of a state $i \in V$ as a random variable $H^i : V \rightarrow {\mathbb {N}} \cup \infty$ given by

$$\begin{aligned} H^i(v) = inf\{n \ge 0: V^i_n(v) = j\} \end{aligned}$$

### Proof

First, we show that $V^i_j$ satisfies Eq. (8). If $i = j$ than $H^i = 0$ by definition and therefore $V^i_j = 0$. If $i \ne j$, than $H^i \ge 1$. According to the Markov property,

$$\begin{aligned} E_i(H^i | V_1 = j) = \frac{1}{\beta } + E_j(H^i). \end{aligned}$$

and

9 $$\begin{aligned} \begin{array}{l} V^i_j = E_j(H^i) = \Sigma _{k \in V} E_i(H^i 1_{V_1 = k}) = \Sigma _{k \in V} E_i(H^i | V_1 = k) P_i (V_1 = k) \\ \quad \,\,\,\,= \frac{1}{\beta } + \Sigma _{k \ne j} \beta V^i_k. \end{array} \end{aligned}$$

Suppose that $y$ is any solution to Eq. (8). Then, for $i = j$, $V^i_j = y = 0$. If $i \ne j$,

10 $$\begin{aligned} \begin{array}{l} y = \frac{1}{\beta } + \Sigma _{k \ne j} \beta y_k = \frac{1}{\beta } + \Sigma _{k \ne j} \beta \big ( 1 + \Sigma _{l \ne j} (\beta y_{k,l}) \big ) = P(H^i \ge 1) \\ \qquad \,+ P(H^i \ge 2) + \dots \end{array} \end{aligned}$$

By repeating this substitution for $y$, in the final term (after $n$ steps), we obtain

11 $$\begin{aligned} \begin{array}{l} y \ge P(H^i \ge 1) + \dots P(H^i \ge n) \end{array} \end{aligned}$$

and, by letting $n \rightarrow \infty$,

12 $$\begin{aligned} \begin{array}{l} y \ge \Sigma _{n=1}^{\infty }P(V^i_j \ge n) = V^i_j . \end{array} \end{aligned}$$

$$\square $$

### Example 1

In the *ladder* graph, as illustrated in Fig. , the inequality in Eq. (8) is sharp. For that, two insights can be concluded. The first one is that each infection path is independent. Namely, if one path is faster or slower it is orthogonal to any other path. The second is that there exists a positive probability realization that the node would be infected by another path than the shortest path. This implies that when one calculates the expected infection time, he would get a lower time than taking only the shortest path.

**Fig. 1:**
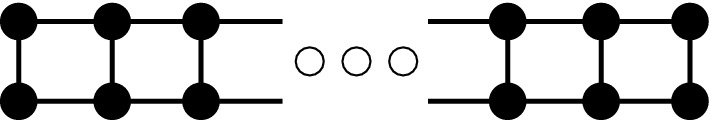
A schematic view of a *ladder* graph

### Corollary 2.1

Given an infection graph with a fixed infection rate $\beta \in (0, 1)$ and recovery rate $\gamma \in (0, 1)$. The infection rate would strictly increase by adding infection paths.

We note that for a single adjacent node, the boundary in Eq. (8) is tight. It can be monotonically relaxed by increasing the number of adjacent nodes, $\gamma$, and $\beta$.

According to Theorem 1 and 2, for $\beta = c$ and $\gamma = 0$, the processes are converging to the same mean. Thus, in the case $\gamma > 0$, the heat spread with diffusion rate $c = \beta$ is an upper boundary of the mean case of the stochastic *SIR* dynamics. This outcome can be obtained by computing the mean infection time from the first infected individual to any other individual in the population. Following this step, one needs to compute the inverse value for this quanta to obtain the mean pandemic spread rate. Nonetheless, using this boundary requires a good approximation of the infection rate ($\beta$). Otherwise, the boundary may be either too high or too low. In the case of the first boundary (Eq. 5), such knowledge is not required.

## Numerical simulations

Based on the proposed theoretical bounds on the pandemic spread, and since these bounds are not tight for some cases, we further investigate them numerically. In this section, we numerically examine the spread dynamics on several graph types. For each graph, we calculate the stochastic *SIR* spread and associated heat spread models.

In particular, $k$-regular graphs, random graphs, and a real-world social interaction graph. We computed the pandemic spread with infection rate of $\beta = 0.07$ and recovery rate of $\gamma = 0.07$. These values were chosen to represent the COVID-19 pandemic [33]. Additionally, according to Theorems 1 and 2, the *maximum* and *mean* diffusion rates are set to be $1$ and $0.07$, respectively.

First, we obtain the connection between the $k$-regularity of a graph and the pandemic spread. In plain English, we computed the mean basic reparation number ($R_0$) of the pandemic. We choose this metric because it is commonly considered to be the proper metric to measure overall pandemic spread [34, 35]. We randomly generated $n=10$ connected, $k$-regular graphs with $|V| = 1000$. The results of this process are presented in Fig. , where the x-axis is the value of $k$ and the y-axis is the mean basic reparation number.

**Fig. 2:**
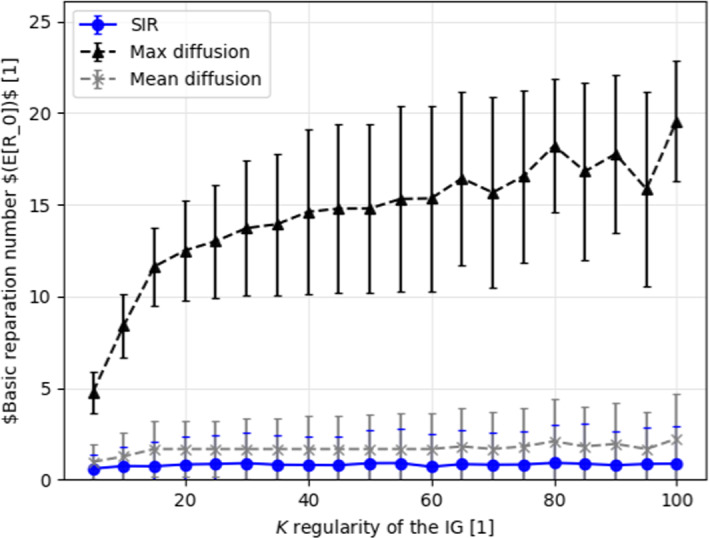
The mean basic reproduction number as a function of the $k$-regularity of the interaction graph. The values provided for the stochastic *SIR* model (blue circles), mean diffusion boundary (gray axis), and maximum diffusion boundary (black triangles). Each sample is shown as mean ± standard deviation for $n = 10$

Since interaction graphs are not necessarily $k$-regular, we computed the mean basic reproduction number for connected, random graphs. The graphs were randomly generated such that each node $v \in V$ has between $3$ and $200$ edges, sampled using a uniform distribution. We generated $100$ samples for graphs at size $|V| = 1000$. The results of this process are presented in Fig. . Where the x-axis is the number of edges in the graph ($|E|$) and the y-axis is the mean basic reparation number.

**Fig. 3:**
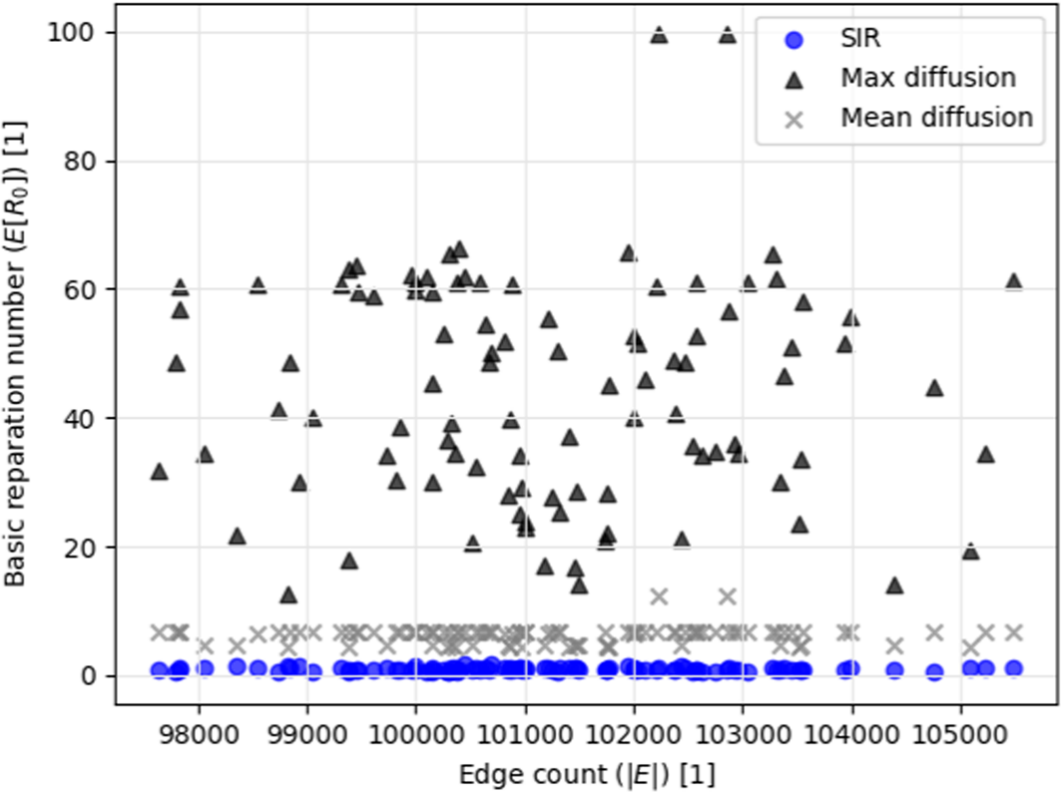
The mean basic reproduction number as a function of the interactions graph’s connectivity (e.g., $|E|$). The values for the stochastic*SIR* model, mean diffusion boundary, and maximum diffusion boundary are provided

The above graphs were constructed synthetically. Thus, a natural question that rise is “does this model words on real-life graphs?”. To answer this question, we tested the model on the Facebook interaction graph. This graph represents the friendships between individuals in the Facebook social platform [36]. For our needs, each individual is set to be a node in the infection graph and each friendship between individuals is assumed to define a possible physical meeting between the individuals and therefore a possible infection route, making it an edge in the infection graph. It contains $|V| = 4039$ nodes and $|E| = 176,468$ edges (1.01% density). Each node $v \in V$ has $44 \pm 52$ neighbors. A histogram of the number of neighbors per node is provided in the supplementary material. We calculated the pandemic spread for the maximum heat spread boundary, the mean heat spread boundary, and the stochastic *SIR* model, as shown in Fig. a–c, respectively.

**Fig. 4:**
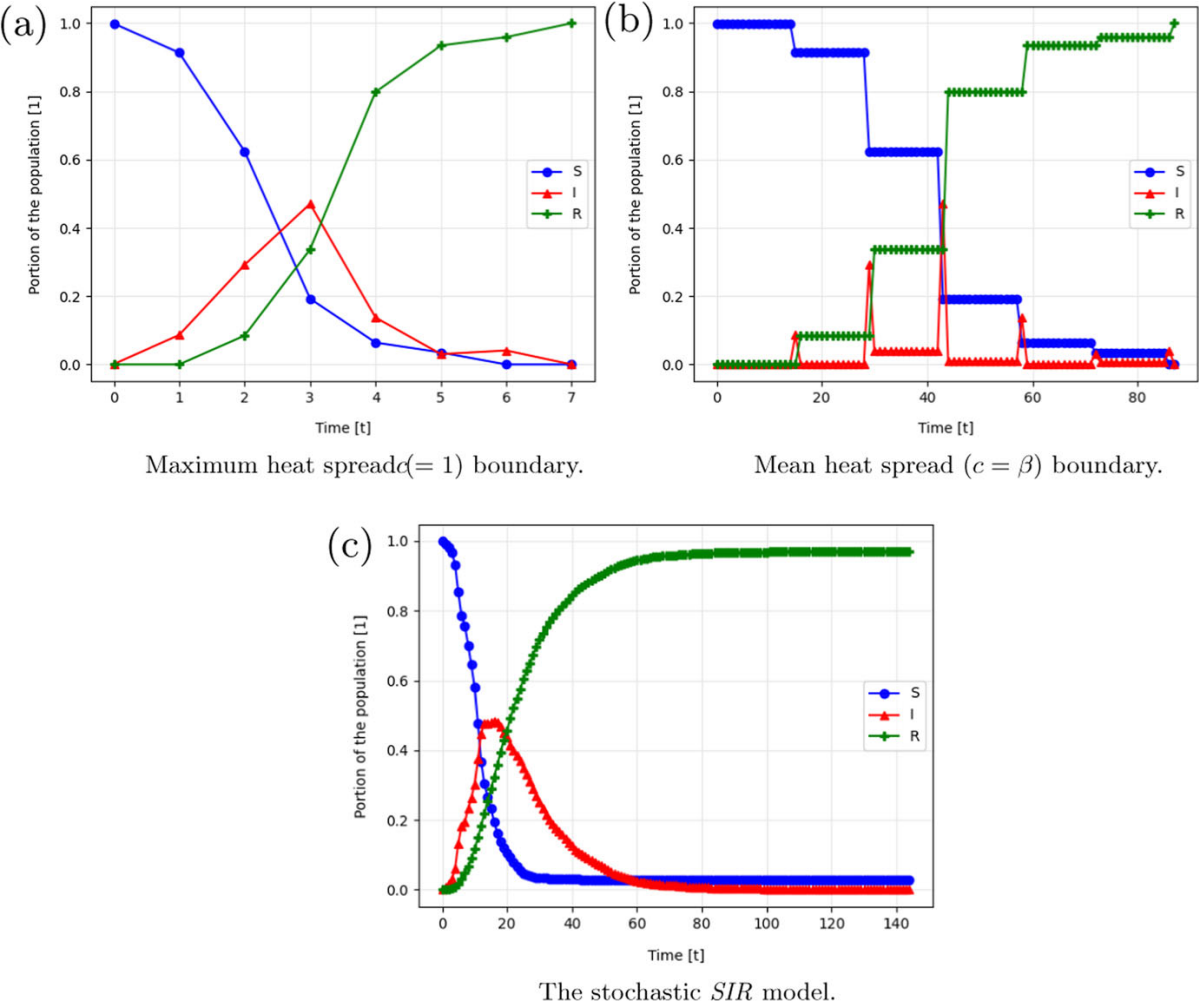
The pandemic spread over time for the Facebook [36] infection graph such that the susceptible, infected, and recovered normalized group sizes are donated by $S$, $I$, and $R$, respectively

## Discussion

Estimating the infection rate is critical information for pandemic management [7, 20]. In this paper, we showed boundaries on the infection rate. By using the heat spread dynamics with different diffusion rates, we learned that the rate is highly dependent on the topology of the interaction graph. The boundaries of a stochastic *SIR* model’s infection rate were assumed to take place on an interaction graph. This provides a better representation of the epidemiological dynamics in a heterogeneous population. Health professionals would benefit from the representation we provide. Since the proposed boundaries are relatively easy to obtain as they require almost no prior data. Specifically, we presented the *worst case* (also called the *maximum case*) and the *mean case* pandemic spread boundaries. This is especially useful at the beginning of a pandemic since acting fast can significantly reduce overall infection [10]. For example, during the COVID-19 pandemic [37], the infection and recovery rates were rapidly update [15, 33, 38–40]. This led to large errors in the estimations of the pandemic’s spread. As a result, policymakers are provided with a distorted image. Hence, the proposed boundaries provide an initial solution. Once more data are gathered, one would be able to both improve the proposed boundaries and use more sophisticated and adjusted models.

The *maximum* heat spread boundary is deterministic tight. Therefore, it cannot be improved. Nonetheless, this case represents a catastrophic scenario where $\beta = 1, \gamma = 0$. This case may cause unnecessary panic and extreme reactions. Obviously, these are not necessarily required to contain the pandemic spread. However, if slightly more information is provided such as the approximation of the infection rate ($\beta$), one can obtain a better approximation of the infection spread rate. Indeed, in such a case, we can use the *mean* heat spread boundary. This boundary provides a tighter approximation to the stochastic *SIR* model. This is done without knowing the recovery rate or anything on the interaction graph, as shown in Fig. 3. Withal, the *mean* heat spread boundary is constituent in providing a mean boundary over the stochastic *SIR*. This is significantly less than the *maximum* heat spread boundary over different levels of connectivity in the population, as shown in Fig. 2. In fact, when applied to the Facebook interaction graph [36], the *maximum* and *mean* heat spread boundaries provided $20$ and $1.66$ times greater pandemic spread rate on average compared to the stochastic *SIR* model, as shown in Fig. 4.

The usage of heat spread as the boundary for the pandemic spread is useful in real settings as one can find the diffusion rate $c$ from local infection spread. For comparison, this method does not work for obtaining the infection rate ($\beta$) and recovery rate ($\gamma$). Therefore, it is faster and more feasible to obtain the heat spread boundaries to the pandemic rather than the *SIR*-based pandemic spread parameters. Thus, while the SIR model is useful, at the beginning of the pandemic where little to no biological and epidemiological knowledge is available, one can first bound the pandemic spread using the diffusion model and later replace it with the SIR one for a more accurate prediction.

A possible future work can be removing the assumption that the interaction graph is static over time. Specifically, one can allow the edges of the graph to change according to some socio-epidemiological logic. This relaxation would lead to a better representation of the pandemic spread in a population. As a result, this can reveal even better boundaries to the pandemic spread.

## Data Availability

All the data that have been used are available online. In the manuscript, we provide links and cite the works that originally presented the data sets.

## Declarations

### Code availability

Upon acceptance, we will publish all the code used in a GitHub repository with technical documentation for easy usage.
